# Impact of Epigenetic Modifications in Airway Diseases. Role of Inflammation, Environmental Factors and Aging

**DOI:** 10.1007/s00408-026-00917-8

**Published:** 2026-07-27

**Authors:** Claudia D’Anna, Caterina Di Sano, Angela Marina Montalbano, Mark GjomarKaj, Fabio Luigi Massimo Ricciardolo, Mirella Profita

**Affiliations:** 1 Institute of Translational Pharmacology, Italian National Research Council, 90146 Palermo, Italy; 2 Department of Clinical and Biological Sciences, University of Turin, Orbassano, Turin, Italy

**Keywords:** Epigenetic, Lung disease, Air pollution, Aging

## Abstract

**Background:**

Airway diseases, including asthma and chronic obstructive pulmonary disease (COPD) are global public health burden due to their high prevalence, morbidity, and mortality. Although these conditions are clinically distinct, they share key pathogenic mechanisms, including chronic inflammation, oxidative stress, and airway remodeling. Continuous exposure of the lungs to environmental agents and pathogens increases their susceptibility to injury and dysregulated immune responses, thereby promoting chronic disease progression. Environmental factors, including cigarette smoke and air pollution, can induce epigenetic changes that act as intermediaries between external insults and disease phenotypes. These epigenetic mechanisms, including DNA methylation, histone modifications, and non-coding RNAs, regulate gene expression without altering the DNA sequence. By modulating chromatin structure and transcriptional activity, they influence immune function, lung homeostasis, and disease susceptibility, thereby contributing to persistent inflammation, airway remodeling, and variability in therapeutic responses.

**Main body:**

Advances in epigenetics have led to the identification of novel biomarkers and potential therapeutic targets, offering new perspectives for diagnosis and treatment. We provide a comprehensive overview of the most recent advances in this field.

**Remarks:**

Current evidence and emerging insightsh discussed in this review might facilitate the understanding of how epigenetic alterations contribute to the biological mechanisms of respiratory diseases and help future research priorities.

**Graphical abstract:**

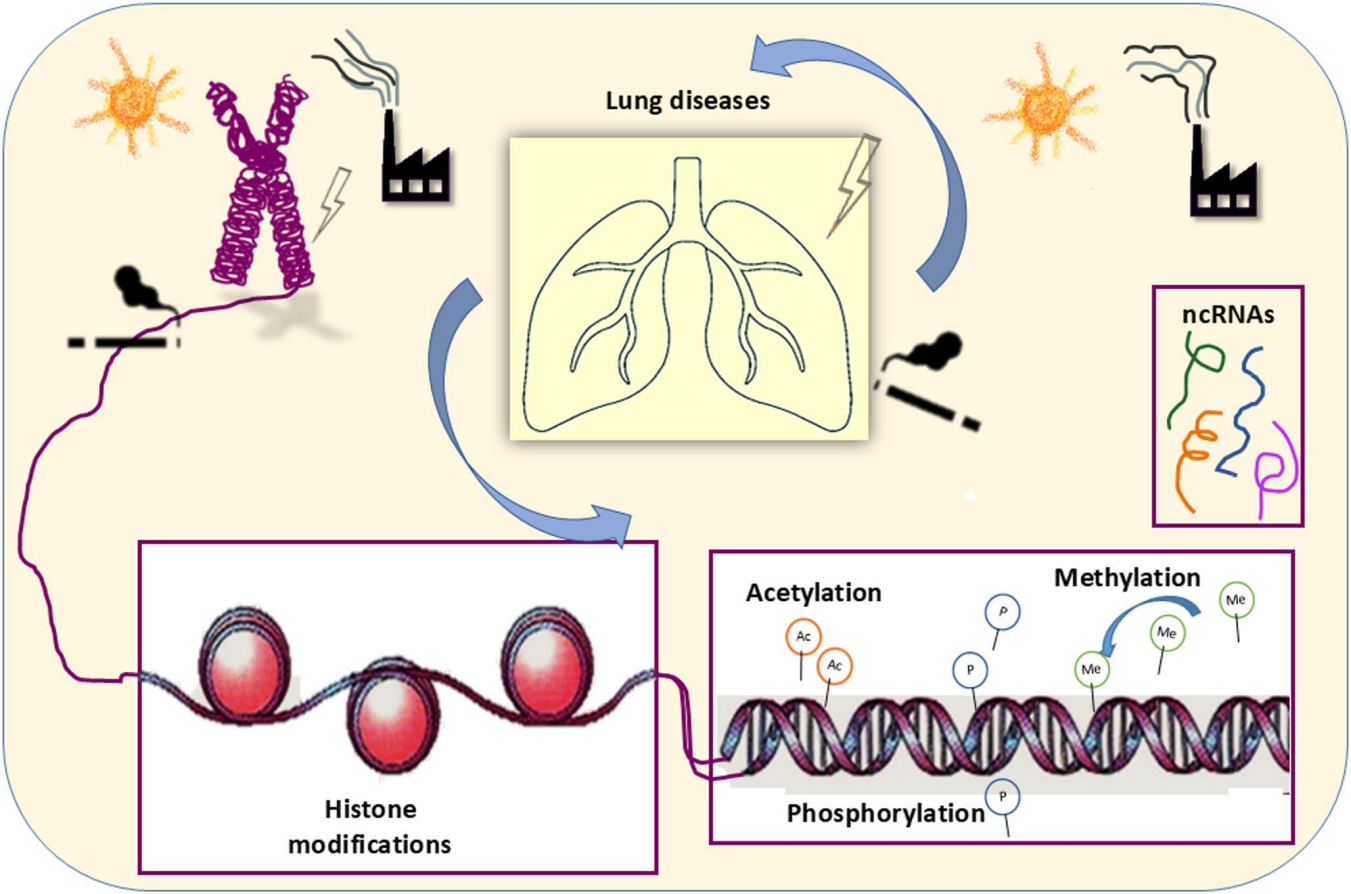

## Introduction

Airway diseases representglobal public health burden due to their high prevalence, significant morbidity, and elevated mortality rates [1]. Although clinically heterogeneous, these disorders share common pathogenic mechanisms, including chronic inflammation, oxidative stress, and airway epithelial remodeling [2, 3]*.* The lungs are continuously exposed to inhaled infectious agents and environmental particulates, such as allergens, dust, and microorganisms, making them particularly susceptible to inflammation and structural damage. The airway innate immune system acts as the first line of defence against these pathogens and irritants [4]. Epithelial, mesothelial, and immunocompetent cells initiate inflammatory cascades via pattern recognition receptors (PRRs), which detect pathogen-associated molecular patterns (PAMPs) and damage-associated molecular patterns (DAMPs), ultimately triggering immune activation. A dysregulated or prolonged response contribute to the progression of chronic respiratory diseases toward more severe lung diseases [5]*.* Environmental exposures, including cigarette smoke, air pollution, and occupational chemicals, can induce stable yet reversible epigenetic modifications, which serve as molecular intermediaries between external insults and disease phenotypes, providing mechanistic insight into how non-genetic factors influence disease susceptibility and progression [6]. Chronic airway diseases show marked clinical and biological heterogeneity. In asthma, phenotypes and endotypes define subgroups with distinct mechanisms, inflammatory pathways, and treatment responses [7]. In this context, epigenetic alterations are unlikely to be uniform across all patients and may instead reflect pheno-/endotype-specific regulatory programs. This appears especially relevant in obesity-associated asthma, where distinct epigenetic mechanisms may contribute to the unique inflammatory and metabolic features of this subtype [8]. A similar perspective may also apply to other chronic inflammatory airway diseases such as COPD, in which phenotypic and molecular heterogeneity is increasingly recognized and may likewise influence disease-associated epigenetic profiles and their clinical significance [9]. Therefore, epigenetic alterations may contribute to the regulation of inflammatory responses in airway diseases, potentially influencing disease progression and therapeutic variability, within the context of distinct disease endotypes rather than a fully uniform biological process. Over the past decade, accumulating evidence has demonstrated that epigenetic mechanisms, including DNA methylation, histone post-translational modifications, and non-coding RNA–mediated regulation, play a pivotal role in regulating pulmonary inflammation and remodeling. They are,dynamically and reversibly involved in gene expression without altering the underlying DNA sequence [10]. By reshaping chromatin architecture, these mechanisms influence transcription factor accessibility, thereby fine-tuning gene expression and enabling cells to rapidly adapt to developmental cues, environmental exposures, and cellular stress. Through these processes, they exert profound effects on immune function, lung homeostasis, and disease susceptibilit [11].

Extracellular vesicles (*EVs) are* important mediators of intercellular epigenetic communication in lung disorders, enable signaling exchanges between epithelial, mesenchymal, endothelial, and immune cells, thereby influencing inflammatory and regulatory pathways relevant to respiratory disease pathogenesis [12]. EVs act as intercellular epigenetic mediators by transferring microRNAs (miRNAs), long non-coding RNAs (lncRNAs), proteins, and lipids, affecting pathological cell–cell crosstalk, aberrant tissue remodeling, and chronic disease progression in inflammatory lung disorders [13–16]*.*

Finally, epigenetic biomarkers are emerging as promising tools for early diagnosis and prognosis, while epigenetic therapies, such as histone deacetylase (HDAC) inhibitors and DNA methyltransferase (DNMT) inhibitors, are currently evaluated in both preclinical and clinical settings, particularly in oncology [17]. Finally, today, epigenetic therapies are increasingly being explored for the treatment of lung diseases [17]. This review provides a comprehensive overview of the role of epigenetic modifications in airway diseases, with a particular focus on asthma and COPD as chronic inflammatory disorders often might be associate with progression of diseases toward lung cancer. It also examines the emerging interplay between epigenetics, aging, and respiratory pathology, highlighting some aspects of current challenges, future directions, and therapeutic opportunities in respiratory epigenetics. Given the breadth and rapidly evolving nature of the topic, this review provides a descriptive overview of recent observations rather than a comprehensive state-of-the-art assessment, with the aim of enhancing understanding of the epigenetic mechanisms involved in chronic inflammatory lung diseases. Owing to the complexity and continuous evolution of this research area, the present overview is not intended to be exhaustive and will inevitably reflect an evolving body of evidence.

## Molecular and Biochemical Hallmark of Epigenetic Modifications

Epigenetic modifications regulate gene activity and integrate environmental signals. Epigenetic mechanisms are heritable changes in gene function without alterations in DNA sequence, primarily mediated by DNA methylation, histone modifications, chromatin remodeling, and non-coding RNAs (ncRNAs) [18]. Chronic exposures can alter the epigenetic landscape of lung cells, leading to aberrant gene expression and contributing to the onset and progression of chronic lung diseases [10]. However, although many epigenetic changes, particularly DNA methylation, have been identified, their underlying molecular mechanisms remain largely unknown.

**Fig. 1:**
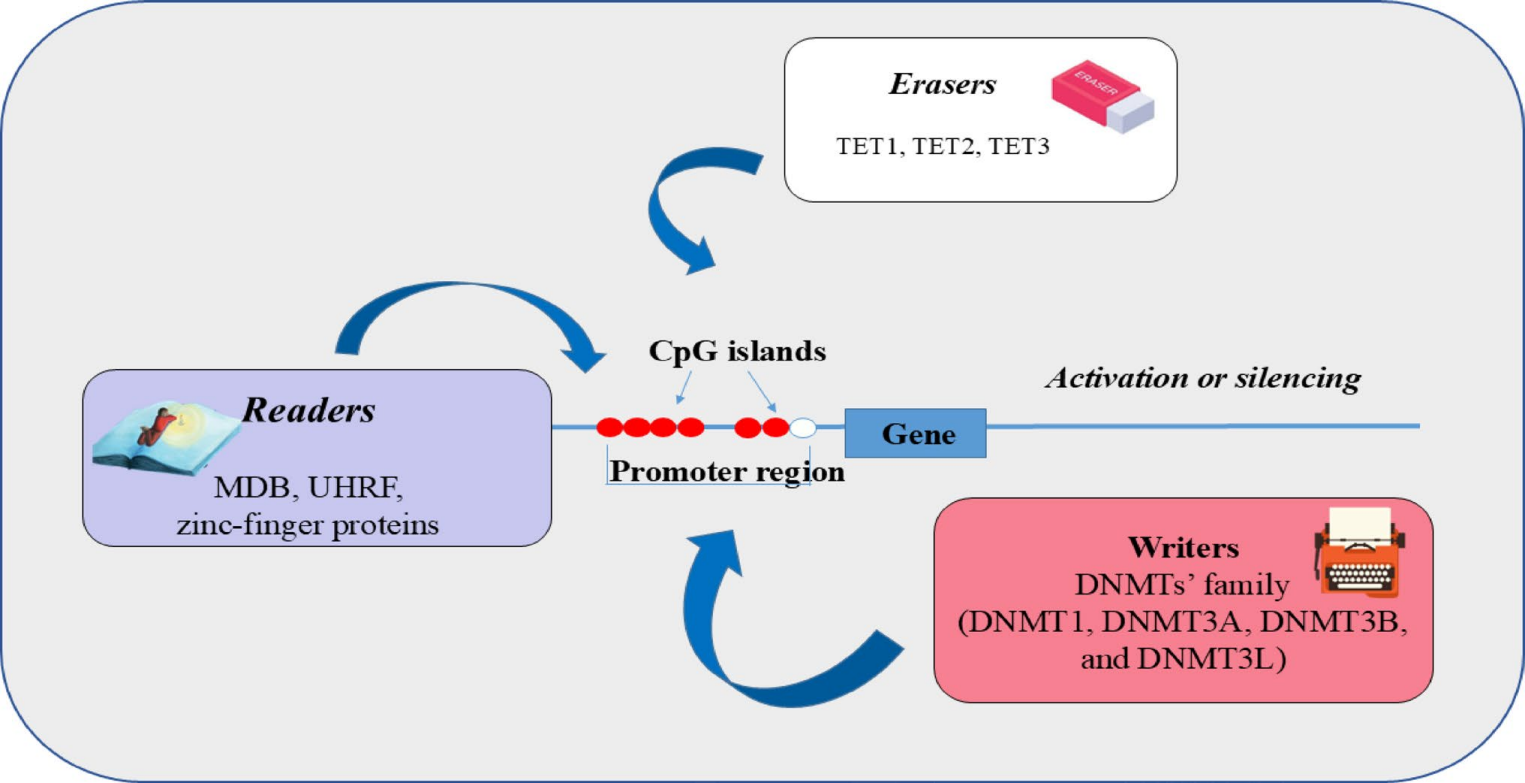
DNA methylation dynamically controls genes through the addition of methyl groups to cytosine residues whitin CpG sites, which are often clustered in CpG islands located in gene promoter region, thereby modulating transcription factor binding. This process is controlled by three main groups of regulators: **a** writers, including DNA methyltransferase family (DNMT1, DNMT3A, DNMT3B, and DNMT3L) which establish and maintain methylation; **b** erasers, including Ten-Eleven Translocation methylcytosine dioxygenase family (TET1, TET2, TET3) which mediate passive or active demethylation via replication-dependent dilution or enzymatic pathways coupled to base excision repair; and **c** readers, including Methyl-CpG Binding Domain proteins (MBD), Ubiquitin-like with PHD and RING Finger domains 1 ( UHRF), and zinc-finger proteins) which recognize methylated DNA and translate these epigenetic marks into changes in chromatin structure and gene expression, thereby contributing to the maintenance of epigenetic stability

DNA methylation modulates gene activity by facilitating or preventing the binding of transcription factors through the addition of a methyl group to cytosines in CpG sequences, catalyzed by DNA methyltransferase (DNMT) enzymes. These CpG sites often cluster in regions known as CpG islands, which are present in ~ 70% of gene promoters and,when methylated, generally suppress gene transcription [19, 20]. Writers, erasers, and readers are three main groups of enzymes that establish, recognize, and remove DNA methylation, enabling dynamic and reversible regulation of gene expression [21]. DNMT family enzymes act as *writers* of DNA methylation. DNMT1 maintains existing methylation during replication, while DNMT3a and DNMT3b establish new methylation in early development. DNMT3L, though catalytically inactive, supports imprinting, retrotransposon silencing, and X-chromosome compaction by stimulating DNMT3a/3b activity, which can also target specific genomic regions via transcription factor or repressor interactions. *Erasers* remove or modify DNA methylation. Passive demethylation occurs gradually during DNA replication when DNMT1 activity is reduced or absent. Active demethylation, which can occur in both dividing and non-dividing cells, involves enzymatic modification of 5-methylcytosine (5mC)—such as oxidation or deamination—followed by replacement through the base excision repair (BER) pathway. *Readers* recognize methylated cytosines and translate these epigenetic marks into functional outcomes, working with writers and erasers to regulate gene expression and maintain epigenetic landscapes. The main families of readers are: (a) MBD proteins (e.g., MeCP2, MBD1–4), which bind methylated CpG sites and recruit repressor complexes, often guiding DNMT1 to hemimethylated DNA; (b) UHRF proteins (UHRF1/2), which target DNMT1 to hemimethylated DNA during replication; and (c) zinc-finger proteins (e.g., Kaiso, ZBTB4, ZBTB38), which bind methylated DNA in a sequence-specific manner to repress transcription (Fig. 1).

Histones form the core of nucleosomes, around which 147 base pairs of DNA are wrapped. Histone post-translational modifications (HPTMs) including methylation, acetylation, and phosphorylation regulate chromatin structure and gene expression in response to environmental signals, influencing development, cellular responses, and diseases including inflammation, and the pathogenesis of inflammatory disease [22]. Histone methylation, catalyzed by histone methyltransferases (HMTs) using S-adenosyl methionine (SAM), can activate (H3K4, H3K36, H3K79) or repress (H3K9, H3K27, H4K20) transcription, while demethylation is carried out by lysine-specific demethylases (e.g., LSD1), often under phospho-signaling control [23, 24].

Histone Acetyltransferase (HATs) and Histone deacetylases (HDACs) control histone acetylation. Histone acetylation is controlled by HATs and HDACs. HATs add acetyl groups to lysines, relaxing chromatin and promoting gene expression, with Class A acting in the nucleus for transcription and Class B transporting new histones from the cytoplasm. Major HAT families include p300/CBP, GNAT, and MYST. HDACs remove acetyl groups, compact chromatin, and repress transcription. In mammals, 18 HDACs belong to two families: zinc-dependent HDACs (Class I: HDAC1–3,8; Class II: HDAC4–7,9–10; Class IV: HDAC11) and NAD⁺-dependent sirtuins (Class III, seven members) [25, 26].

**Fig. 2:**
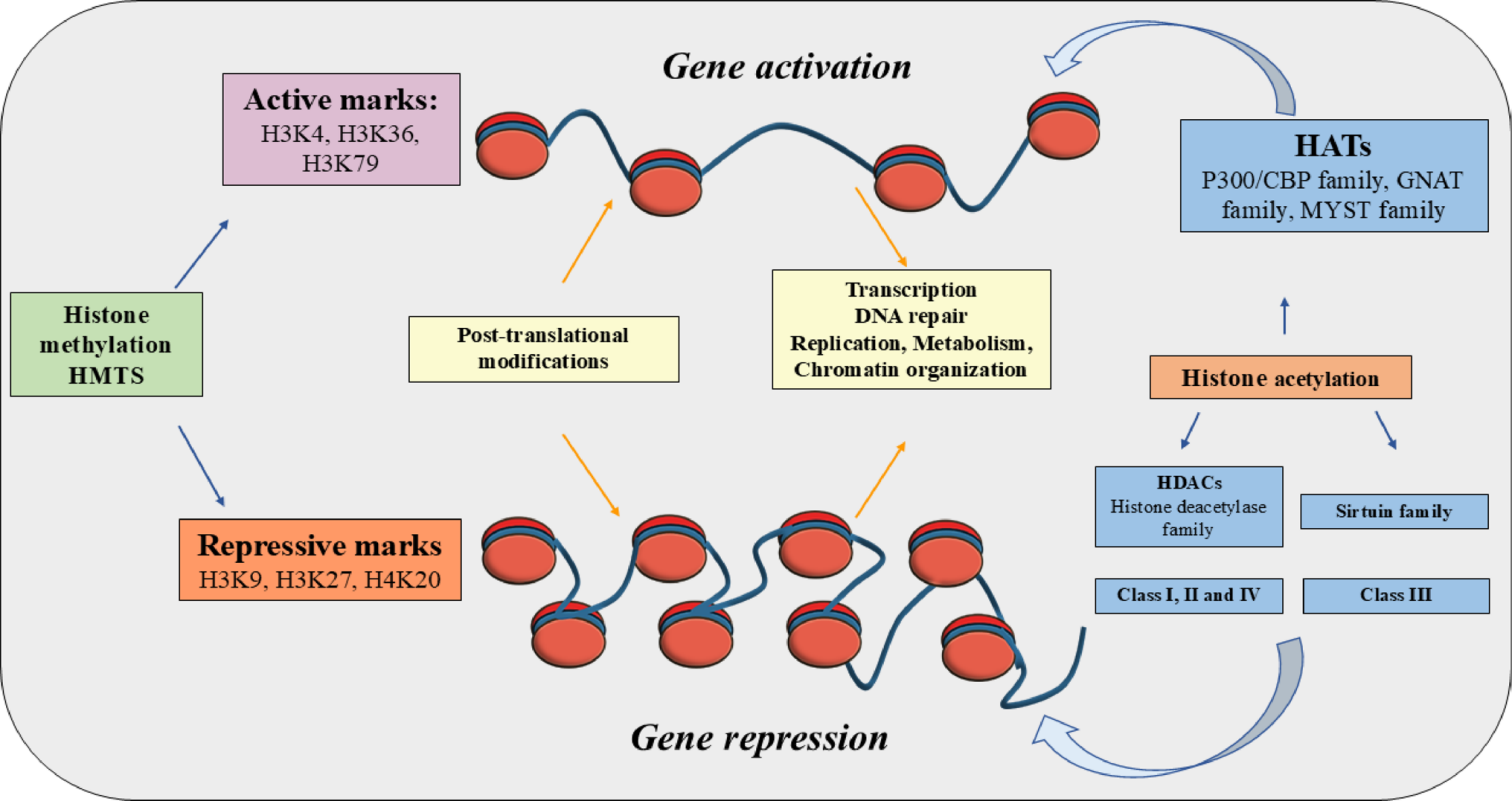
Histone post-translational modifications in chromatin regulation. Histones package DNA into nucleosomes and undergo dynamic modifications such as methylation, acetylation, and phosphorylation. These changes are regulated by specific enzymes and influence chromatin structure and gene expression. Histone methylation can either promote or repress transcription depending on the modified residue and its genomic contex, whereas acetylation, mediated by Histone Acetyltransferase (HATs), generally enhances chromatin accessibility by weakening histone–DNA interactions; conversely, Histone deacetylases (HDACs) reverse this effect by removing the acetyl group. Histones undergo additional modifications such as phosphorylation, ubiquitination, SUMOylation, and acylations (e.g., crotonylation, butyrylation), which further contribute to the dynamic regulation of chromatin structure and function

Histone phosphorylation is a reversible modification in which Protein Kinase A (PKA), Cyclin-Dependent Kinases, and Serine/Threonine Kinase (ATR) add phosphate groups to serine, threonine, or tyrosine residues on histone tails, often in response to DNA damage. This recruits DNA damage response proteins and regulates transcription, cell cycle, and apoptosis, while phosphatases such as Protein Phosphatase 2 remove the phosphate to restore chromatin. Other post-translational modifications, including propionylation, butyrylation, crotonylation, and SUMOylation, also affect transcription, DNA repair, replication, metabolism, and chromatin organization [27, 28]. Phosphorylation of H2AX at serine 139 by ATM, ATR, and DNA-dependent protein kinase produces γH2AX, a key marker of double-strand DNA breaks that recruits repair proteins in the inflammatory processes [29]. The diverse roles of histone modifications make them promising therapeutic targets and clinical biomarkers, emphasizing the importance of understanding histone regulation in health and disease (Fig. 2).

NcRNAs are short RNA molecules that do not encode proteins They include microRNAs (miRNAs), long non-coding RNAs (lncRNAs), and PIWI-interacting RNAs (piRNAs) [30]. These molecules modulate gene expression by influencing chromatin organization, transcriptional regulation, and post-transcriptional control [31].

MiRNAs are key regulators of DNA methylation patterns, primarily through the modulation of DNA methyltransferases (DNMTs). They also affect histone modification states by targeting enzymes involved in histone acetylation and methylation, thereby contributing to chromatin remodeling and transcriptional regulation. For example, members of the miR-29 family target DNMTs, leading to altered methylation patterns at specific genomic loci [32]. In addition, miRNAs regulate gene expression post-transcriptionally by guiding the RNA-induced silencing complex (RISC) to complementary sequences, typically located within the 3′ untranslated region (3′UTR) of target mRNAs. Binding of the miRNA–RISC complex leads to translational repression and/or target mRNA degradation, depending on the degree of sequence complementarity, with mRNA destabilization representing the predominant mechanism of miRNA-mediated gene silencing in mammalian cells [33, 34].

LncRNAs, such as HOX transcript antisense RNA (HOTAIR) and X-inactive specific transcript (XIST), function as epigenetic regulators. HOTAIR influences DNA methylation through interactions with DNMTs, whereas XIST modulates histone modifications, leading to gene silencing. Coversely, both DNA methylation and histone acetylation can regulate ncRNA expression, resulting in either transcriptional upregulation or downregulation [35].

**Fig. 3:**
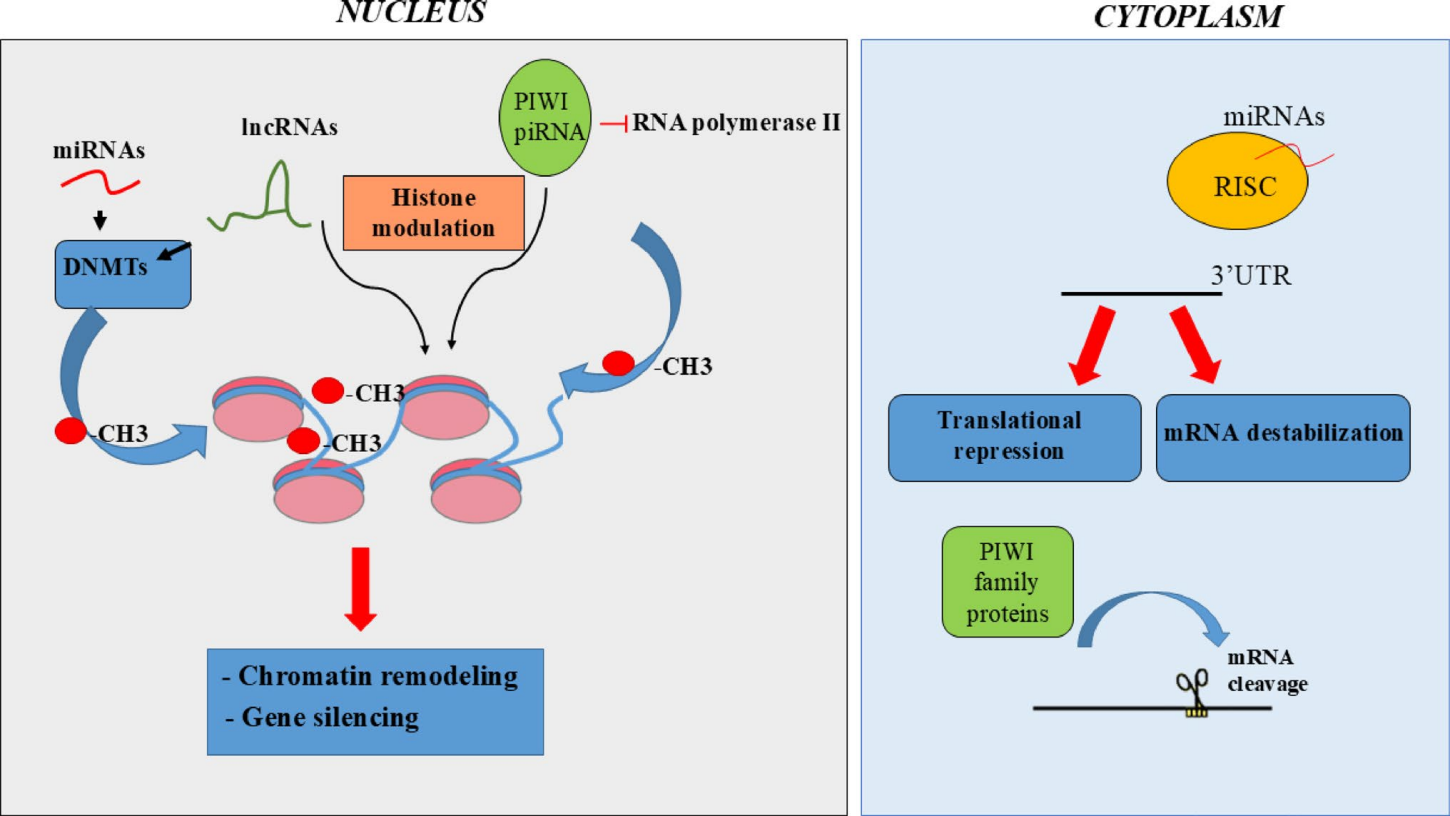
Non-coding RNAs in epigenetic regulation. MicroRNAs (miRNAs), long non-coding RNAs (lncRNAs), and PIWI-interacting RNAs (piRNAs) regulate gene expression through epigenetic and post-transcriptional mechanisms. MiRNAs promote target mRNA degradation or translational repression and modulate DNA methylation and histone modifications. LncRNAs regulate the activity of epigenetic modifiers, whereas PIWI/piRNA complexes contribute to chromatin regulation and mediate sequence-specific target mRNA cleavage

PIWI/piRNA complexes play an essential role in chromatin regulation, including DNA methylation, histone methylation, histone acetylation, and histone ubiquitination. PiRNAs promote the recruitment of HP1α protein to specific genomic loci, contributing to the repression of RNA polymerase II–dependent transcription. Additionally, PIWI family proteins, guided by piRNAs, can cleave target mRNAs, thereby exerting an additional post-transcriptional regulatory function. nder extreme inflammatory conditions in the lung, dysregulation of PIWI/piRNA-mediated mechanisms may contribute to aberrant gene expression and inflammatory disease progression [36] (Fig. 3).

## Asthma and Epigenetic Modifications

Asthma is one of the most prevalent chronic respiratory diseases, typically originating early in life and characterized by airway inflammation and reversible airflow obstruction. Its clinical presentation is heterogeneous, with symptoms including wheezing, shortness of breath, cough, and chest tightness. This marked heterogeneity highlights the limitations of current symptom-based therapies and underscores the need for a deeper understanding of the underlying pathogenic mechanisms. The onset and progression of asthma arise from complex interactions among genetic susceptibility, environmental exposures, and immune maturation during early life. Moreover, impairment of the epithelial barrier and dysregulation of immune responses further increase susceptibility to external insults, thereby contributing to disease development and persistence [37, 38].

The interplay of genetic, environmental, and socioeconomic factors contributes to the heterogeneity and variability of asthma. Epigenetic processes, including DNA methylation, histone modifications, and ncRNAs, are increasingly recognized as key regulatory mechanisms, as they link environmental exposures to molecular and cellular responses. These mechanisms influence critical immune and inflammatory pathways by modulating cytokine expression and cell differentiation, thereby providing new insights into the pathogenesis of asthma and offering promising perspectives for the development of more targeted therapeutic strategies [38, 39].

DNA methylation changes have been widely studied in asthma, particularly in genes related to T helper (Th) cell differentiation and immune response [40]. In a mouse model, it was observed that DNMT1 in dendritic cells orchestrates Th2 polarization through the silencing of Il12b and the stabilization of TIM4, positioning DNMT1 inhibitors and TIM4-targeted therapies as novel strategies to rebalance Th1/Th2 responses in asthma [41].

The risk of developing allergic disorders later in life is already detectable at birth, suggesting the involvement of epigenetic mechanisms in childhood asthma. Differences in DNA methylation of Th2-related genes, such as IL4 and GATA3, have been observed between children who develop asthma early in life and those who develop it later. Increased methylation of GATA3 CpG sites at positions − 2211/ − 2209, located within a NF-κB binding site, has been shown to inhibit transcription factor binding in the human Jurkat T cell line at birth and is associated with a reduced risk of asthma at 3 years of age [42]. STX4 influences childhood asthma risk through its role in CD4 + and CD8 + T cell.

subsets and is considered a promising candidate gene for precision medicine approach. Its expression is regulated by DNA methylation at key CpG sites, with higher methylation levels associated with a reduced risk of asthma [43].

Epigenetic regulation plays a key role in IL-33–mediated immune responses. IL-33, a member of the IL-1 family, activates NF-κB and MAP kinases and induces Th2-associated cytokines, including IL-4, IL-5, and IL-13, thereby contributing to asthma pathogenesis. In primary bronchial epithelial cells from asthmatic and non-asthmatic individuals, IL-33 DNA methylation patterns were associated with asthma status, with lower methylation levels associated with increased CCL26 expression and higher lung eosinophil counts in asthmatic patients [44]. In particular, the DNA methylation status of IL-33 gene promoter region has been closely associated with the onset of pediatric asthma, with specific hypomethylation at CpG_8 significantly correlating with with Th2 cytokine expression, rs4742170 SNP gene variations [45].

Furthermore, abnormal methylation of the FOXP3 gene, which regulates regulatory T-cell (Treg) cell activity, has been associated with Treg dysfunction and dysregulated immune responses in asthma [46]. Abnormal methylation of HLA-DMB and FOXP3 in asthma patients impairs T cell maturation and Treg function, respectively, contributing to dysregulated immune responses and asthma pathogenesis. In asthma, thousands of DNA methylation–gene expression associations have been identified, highlighting the role of epigenetic regulation in the disease [47, 48].

Alongside DNA methylation, histone acetylation contributes to the epigenetic landscape of asthma. The expression and activity of HATs and HDACs are altered during pulmonary inflammation, with opposing effects: HATs promote gene expression, whereas HDACs suppress it [13, 49]. In asthmatic patients, increased HAT activity and, in particular, decreased HDAC2 expression in airway cells are strongly associated with enhanced transcription of pro-inflammatory genes, thereby playing a crucial role in the progression of this disease [40].

P300 and KAT8 are HATs that mediate hyperacetylation of H3K9 and H3K14, leading to increased production of IL-4 and IL-33, both proinflammatory cytokines involved in the initiation and progression of asthma [50]. In contrast, HDACs can exert anti-inflammatory effects. For example, the transcription factor Ets2 epigenetically suppresses TLR-induced IL-6 production, a key pro-inflammatory cytokine in asthma, by promoting HDAC1-mediated deacetylation of the IL-6 promoter in both human and murine macrophages. This mechanism highlights Ets2–HDAC1 signaling as a potential therapeutic target for inflammatory diseases [51].

HDAC8 activation increases airway hyperresponsiveness (AHR) in animal models of allergic asthma by enhancing the interaction between HDAC8 and Galectin-3 (Gal-3) and promoting M2 macrophage polarization [52]. Furthermore, in a mouse model of asthma, treatment with the HDAC8 inhibitor PCI-34051 completely reversed histological and cellular changes associated with airway inflammation and remodeling by regulating miR-381-3p and its downstream target gene TGF-β3, thereby alleviating airway hyperresponsiveness [53, 54]. Recent studies have expanded our understanding of the role of histone acetylation, a classical mark of epigenetic mechanism in regulating the physiological processes of a variety of cell types involved in the pathogenesis of asthma, including immune cells (e.g., CD4 + T cells, macrophages) and structural lung cells (e.g., airway epithelial cells, pulmonary fibroblasts) [55]. Modulation of histone acetylation levels in the immune system through dietary interventions may protect against the development of asthma and other allergic diseases. Additionally, targeting the enzymes that regulate histone acetylation in structural lung cells and local immune cells could offer promising therapeutic strategies for asthma [56]. Despite some methodological challenges, analysis of histone acetylation levels may improve asthma diagnostics. Interestingly, differential patterns in 15 of 32 genes observed in airway epithelial cells (AECs) from subjects with eosinophilic versus no-eosinophilic asthma corresponded to those reported in human lung fibroblasts and were associated with specific histone modifications, including H3K9 acetylation and H3K27 trimethylation [54].

SIRT6 overexpression modulated TGF-β1–induced epithelial–mesenchymal transition (EMT) markers and cellular behaviours. Upregulated during airway remodeling, SIRT6 regulated EMT in bronchial epithelial cells via the Smad3 and c-Jun pathways, suggesting it as a potential therapeutic target in asthma. Specifically, SIRT6 overexpression suppressed cell migration and proliferation, reduced Smad3 phosphorylation, decreased H3K9 acetylation, and inhibited c-Jun transcription. It also reversed TGF-β1–induced changes in E-cadherin, N-cadherin, vimentin, α-SMA, and MMP-9 in 16HBE cells [57]. Furthermore, SIRT6 is an epigenetic regulator that governs IL-17A pathogenicity in severe asthma. It affects airway inflammation and remodeling in asthmatic mice, implicating SIRT6 as a potential therapeutic target for severe asthma [58].

Allergic diseases are triggered by environmental allergens. Histone acetylation links these exposures to immune responses and plays a central role in asthma, food allergies, and atopic dermatitis, thereby constituting a promising therapeutic target. In a murine model of OVA-induced allergic inflammation, proteomic analyses revealed alterations in 154 proteins and 68 acetylation sites, underscoring the importance of epigenetic modifications in asthma [59]. MiRNAs and lncRNAs regulate immune responses and epithelial function in asthma, controlling genes involved in inflammation, immune activation, and barrier integrity. Differential lncRNA expression has been observed in CD4⁺ and CD8⁺ T cells from both asthmatic patients and mice, highlighting their role in asthma pathogenesis and their potential role of biomarkers or therapeutic targets [60]. Ll ncRNA fantom3_9230106C11 was found to be significantly reduced in CD4⁺ T cells and Th2 cells in an acute asthma model [61]. Another notable example is MALAT1, which regulates CTLA-4 expression through a miR-155 “sponge” mechanism, thereby influencing the Th1/Th2 balance in CD4⁺T cells. These findings suggest that interactions between lncRNAs and miRNAs play an important role in asthma pathogenesis and may serve as potential therapeutic targets as well as diagnostic biomarkers [62]. In recent years, circRNAs, a class of ncRNAs, have emerged as important regulators of gene expression owing to their covalently closed-loop structure, which confers remarkable stability. Among them, mcircRasGEF1B is induced by lipopolysaccharide (LPS) and has attracted attention for its role in regulating inflammatory responses. They contribute to immune modulation by regulating the stability of intercellular adhesion molecule-1 (ICAM-1) mRNA, thereby influencing inflammatory and immune responses. These findings underscore the role of circRNAs in immune-related diseases as asthma and their potential as diagnostic biomarkers and therapeutic targets [63].

Although specific studies are still limited, circRNAs are hypothesized to play an important regulatory role in T cell–mediated inflammation in asthma. Supporting this hypothesis, one research group analyzed circRNA expression in CD4⁺ T cells from patients with asthma and found that several circRNAs are differentially expressed compared with those in healthy individuals. Hsa_circ_0005519 has been shown to bind hsa-let-7a-5p through a miRNA “sponging” mechanism, thereby regulating its activity. This interaction influences the production of cytokines, including IL-13 and IL-6, which are key mediators of airway inflammation. Consequently, hsa_circ_0005519 contribute to T cell–mediated inflammatory processes in asthma, highlighting the role of circRNAs in the molecular mechanisms of diseases underlining their potential as biomarkers or therapeutic targets [64]. This study provides new insight into the pathogenesis of asthma, highlighting the potential role of ncRNAs, particularly circRNAs, in the regulation T cell–mediated inflammatory responses. By showing how circRNAs can influence cytokine secretion and immune signaling pathways, these findings broaden our understanding of the molecular mechanisms underlying asthma and suggest that circRNAs may serve as novel biomarkers or therapeutic targets. During asthma exacerbations, patients exhibited reduced serum levels of miRNA-126a, miRNA-16, and miRNA-21 with some of these alterations correlating with lung function and airway inflammation. CD4⁺IL-4⁺ cells were increased, whereas regulatory Tcells and eosinophil progenitors were associated with better asthma control. Serum miRNAs levels negatively correlated with pro-inflammatory Tcell cytokines.

suggesting that epigenetic dysregulation impacts lung function, airway inflammation, and T cell mediated cytokine expression [65]

In asthma, miRNAs are key regulators of disease pathophysiology and gene expression (Table 1). These ~ 22-nucleotide ncRNAs act as epigenetic modulators at the post-transcriptional level, targeting messenger RNA (mRNA). They are also emerging as mediators of inter-tissue communication, particularly between adipose tissue and the lung, within EV-mediated ncRNA signaling pathways [14, 34].

**Table 1:** Major epigenetic modifications and their impact on asthma pathogenesis

Epigenetic mechanism	Target/factor	Functional consequence in asthma	Refs
DNA methylation	DNMT1	Promotes Th2 polarization and allergic inflammation	[41]
DNA methylation	IL4, GATA3	Influences Th2 differentiation and childhood asthma susceptibility	[42]
DNA methylation	STX4	Modulates CD4⁺ and CD8⁺ T-cell function	[43]
DNA methylation	IL-33 promoter	Enhances Th2 responses and asthma severity	[45]
DNA methylation	FOXP3, HLA-DMB	Loss of immune tolerance and chronic inflammation and immune dysregulation	[47, 48]
Histone acetylation	HATs (P300, KAT8)	Upregulates IL-4 and IL-33 expression, promoting airway inflammation	[50]
Histone deacetylation	HDAC2	Increased transcription of pro-inflammatory genes	[40]
Histone deacetylation	HDACs	Anti-inflammatory effect	[51]
Histone modification	SIRT6	Potential therapeutic target in severe asthma. Regulates EMT	[57, 58]
lncRNA	fantom3_9230106C11	Implicated in Th2-mediated inflammation	[61]
lncRNA	MALAT/mir155	CTLA-4 regulation	[62]
circRNA	mcircRasGEF1B	Modulates inflammatory and immune responses	[63]
circRNA	hsa_circ_0005519	Promotes IL-13 and IL-6 production and T-cell inflammation	[64]
miRNAs	miR-126a, miR-16, miR-21	Associated with lung function, airway inflammation, and disease control	[65]

## COPD and Epigenetic Modifications

Cigarette smoke is a major environmental driver of epigenetic alterations, inducing persistent changes in DNA methylation that modulate inflammatory responses and increase susceptibility to COPD. Mechanistically, reactive compounds present in cigarette smoke, including arsenic, formaldehyde, and nitrosamines, can induce DNA damage and trigger the recruitment of DNMTs during repair processes, resulting in localized changes in CpG methylation [66]*.* DNA methylation plays a pivotal role in the inflammatory processes and pathophysiology of COPD, acting as a dynamic and potentially reversible epigenetic modification across multiple disease-relevant tissues. It represent a valuable biomarker of disease and a promising therapeutic target [67, 68]*.* Epigenome-wide association studies (EWAS) have identified robust smoking-related methylation signatures across multiple tissues, including hundreds of differentially methylated CpG sites [66]*.* DNA methylation at AHRR locus emerges as a key marker, showing significant variability and partial reversibility after smoking cessation, supporting the functional role of these epigenetic modifications in disease pathways [69]*.* Tissue-specific analyses provide direct evidence of epigenetic involvement in COPD. A total of 1,155 differentially methylated positions were identified, revealing a complex epigenetic landscape that may contribute to dysregulated inflammatory responses. Notably, genes such as ZDHHC14, NUB1, NLRP3, and ZNF322 exhibited both hypermethylation and hypomethylation patterns [70]. Cigarette smoke increases METTL3 and IGF2BP2 expression in alveolar macrophages. METTL3-mediated m6A RNA methylation stabilizes CXCL8 mRNA through IGF2BP2, promoting neutrophil recruitment, and stabilizes ICAM-1 mRNA in endothelial cells, enhancing neutrophil adhesion and transmigration. This m6A-dependent mechanism amplifies neutrophilic inflammation, revealing a novel METTL3/IGF2BP2/m6A methylation pathway that coordinates macrophage–endothelial interactions in COPD and represents a potential therapeutic target [71]*.* In mouse models (C57BL/6 and A/J), prolonged in vivo exposure to cigarette smoke induces global epigenomic remodeling in lung tissue, characterized by thousands of differentially methylated regions that predominantly affect genes involved in inflammatory and immune pathways [72]*.* High-resolution approaches, including reduced representation bisulfite sequencing (RRBS) and oxidative bisulfite-based methods, have further demonstrated that cigarette smoke exposure affects both 5-methylcytosine and 5-hydroxymethylcytosine at specific genomic loci, revealing a multilayered reprogramming of the epigenetic landscape rather than a uniform global change [73]*.* Many of these epigenetic alterations are specifically mediated by DNMT1, which promote promoter hypermethylation and transcriptional repression of key genes. Notable targets include COX-2, involved in inflammation and endothelial apoptosis, as well as HSH2D, SNX10, CLIP4, and TYK2, which play central roles in immune signaling pathways [74]*.* The PTEN gene, acting as tumor suppressor, is frequently hypermethylated in lung tissue from patients with COPD and smokers [75]*.* Oxidative stress and hypoxia enhance methyl donor availability and activate antioxidant and inflammatory transcriptional programs, collectively linking methylome remodeling to xenobiotic response pathways and disease susceptibility in mouse exposed to cigarette smoke [76].

Epigenetic changes may represent potential prognostic markers of COPD, as hypermethylation of CpG sites in the PI3KCD gene has been associated with increased mortality risk, while differential methylation of SERPINA1, which encodes α1-antitrypsin and modulates pulmonary inflammatory responses, correlates with immune activity [77]. Reduced global DNA methylation, commonly assessed via LINE-1 elements, further suggests a contribution to genomic instability and disease progression. In addition to cigarette smoke, other environmental exposures can shape the lung epigenome. In women with COPD, chronic biomass smoke exposure was associated with reduced methylation of the PRSS23 gene (cg23771366), demonstrating that inhaled particulates beyond tobacco can drive locus-specific epigenetic alterations [77]*.* Consistent with these findings, methyl-capture sequencing in COPD cohorts revealed cumulative exposure-dependent genome-wide methylation changes in response to PM2.5 exposure [78]*.* These findings suggest that epigenetic remodeling links environmental exposures to biological responses, while the reversible nature of DNA methylation highlights its potential role as a therapeutic target for restoring gene expression and reducing chronic inflammation in COPD.

Histone modifications, together with DNA methylation, constitue a key epigenetic network in COPD, regulating genes involved in inflammation, tissue remodeling, and cellular aging [79]*.* Dysregulation of HDACs, particularly HDAC1 and HDAC2, in skeletal muscle further underscores the systemic nature of chronic COPD, contributing to sarcomere disorganization, mitochondrial dysfunction, and muscle wasting (cachexia) [80]. Skeletal muscle dysfunction is now recognized as a major extrapulmonary manifestation of COPD, driven by complex molecular alterations, including epigenetic dysregulation and impaired proteostasis [81]*.* Oxidative and nitrosative stress induced by CS and environmental pollutants plays a pivotal role in reducing HDAC activity through post-translational modifications, including nitrosylation and carbonylation, leading to enzymatic inactivation and proteasomal degradation [82]*.* The loss of HDAC2 function amplifies inflammatory signaling and contributes to corticosteroid resistance, which, as above mentioned, represent a hallmark of COPD pathophysiology [83]*.* Therapeutic strategies targeting histone acetylation show promising potential. Pharmacological agents, such as thiols, can restore HDAC activity and improve corticosteroid sensitivity [84]*,* while natural compounds such as curcumin can partially restore HDAC2 function and attenuate inflammatory responses in COPD patients [85]*.* Although pan-HDAC inhibitors such as trichostatin A are primarily used as experimental tools, they have contributed to elucidating the role of epigenetic regulation in COPD [82]. The development of selective HDAC2 activators and HAT inhibitors is a promising strategy to restore epigenetic balance and improve cellular function in COPD [86].

Beyond acetylation, histone methylation has gained increasing attention, particularly trough the protein arginine methyltransferases (PRMTs), which regulate both inflammatory and regenerative processes [87]*.* PRMT7 is a key modulator of innate immune responses and is upregulated in monocytes from COPD patients, where it promotes arginine methylation at enhancer regions, including the Rap1a gene, thereby enhancing monocyte adhesion and infiltration into lung tissue. Consistently, PRMT7 deficiency in experimental models reduces monocyte and macrophage recruitment and protects against cigarette smoke–induced lung injury, underscoring its pathogenic role in COPD progression [88]. In contrast, coactivator-associated arginine methyltransferase-1 (CARM1/PRMT4) plays a protective role in airway epithelial cells. Reduced CARM1 expression is associated with impaired epithelial regeneration, increased cellular senescence, and defective tissue repair, whereas a normal CARM1 activity maintains epithelial integrity and homeostasis [89]*.* These findings suggest that PRMTs exert context-dependent effect in COPD, contributing both to inflammatory responses and to the regulation of tissue repair.

Beyond acetylation and methylation, other histone modifications contribute to shaping the COPD epigenome. Phosphorylation of histone H3 activates NF-κB–dependent pro-inflammatory genes expression and is regulated by stress-responsive pathways, including p38 MAPK, which are triggered by oxidative stress and environmental stimuli [90]*.* The coordinated interplay among histone modifications represents a central mechanism that integrates multiple epigenetic signals, thereby influencing gene expression, chromatin dynamics, and ultimately driving inflammation and tissue remodeling in COPD [13]*.* Both early studies on histone acetylation and recent insights into PRMT-mediated methylation and epigenetic crosstalk highlight chromatin remodeling as a key mechanism in COPD pathogenesis. Targeting specific HDACs, PRMTs, and related signaling pathways represent a promising strategy to restore epigenetic balance, reduce chronic inflammation, and promote tissue repair. In the future, the identification of robust epigenetic biomarkers may enable personalized therapeutic approaches, improving patient stratification and clinical outcomes.

NcRNAs regulate key processes in COPD, including airway remodeling, chronic inflammation, oxidative stress, cellular senescence, and apoptosis, acting within interconnected networks that integrate environmental and intracellular stress signals [91]. MiRNAs play a key role in the post-transcriptional regulation of gene expression and are involved in the main pathogenic mechanisms of COPD, as demonstrated by both experimental and clinical studies. In particular, the Let-7 family has been shown to enhance IL-6 secretion in human bronchial epithelial cells and promote fibroblast differentiation into myofibroblasts, thereby contributing to airway remodeling in COPD patients [92]. Clinical evidence highlights the relevance of miRNA dysregulation in COPD, with altered circulating and tissue-specific miRNA signatures being associated with disease severity, inflammation, and lung function decline [93]. MiRNA-mediated effects are modulated by environmental factors. For example, cadmium exposure suppresses miR-30 in bronchial epithelial cells, leading to upregulation of the EMT regulator SNAIL and promoting structural changes similar to those observed in lung of smokers, as demonstrated by in vitro and murine studies [94]. In alveolar macrophages and BEAS-2B epithelial cells, the downregulation of miR-181 and miR-218 enhances NF-κB activation and cytokine release, while in murine models, elevated miR-155 amplifies immune responses and impairs lung function, reproducing the airflow limitation characteristic of COPD [95, 96]. Furthermore, recent evidences highlight miR-155 as a key pro-inflammatory regulator in COPD, linking NF-κB activation to increased cytokine production [97].

Specific miRNAs, such as miR-34a and miR-570, regulate cellular senescence and regenerative capacity in bronchial epithelial cells. In BEAS-2B cells and primary small airway epithelial cells from COPD patients, miR-34a is upregulated by oxidative stress or cigarette smoke extract exposure, targeting SIRT1 and SIRT6 and promoting senescence pathways [98, 99]. In vitro and in vivo studies, including cigarette smoke–exposed murine models, miR-34a and miR-570 converge on SIRT-dependent and inflammatory pathways, interact with profibrotic signaling such as TGF-β, and thereby impair epithelial repair, contributing to airway remodeling and tissue damage in chronic lung diseases [100]. All these findings highlight how miRNAs act as modulators able to balance inflammation, structural remodeling, and stress responses, maintaining epithelial homeostasis in COPD lungs [91].

Circular RNAs (circRNAs) modulate COPD pathogenesis by acting as miRNA sponges or scaffolds for regulatory proteins. CircRNA_0026344 protects alveolar epithelial cells from cigarette smoke extract–induced apoptosis by sequestering miR-21 and preserving PTEN function in asthma and COPD [101, 102]. Conversely, circBBS9, circ_0026466, and circ_0006872 promote apoptosis and inflammation via interactions with miR-103a-3p, miR-153-3p, and miR-145, thereby modulating NF-κB signaling in COPD models [91, 103]. CircSAV1 acts as a scaffold that enhances IREB2 translation and ferroptosis, whereas circXPO1 and circFOXO3 amplify inflammatory responses through TGF-β and NF-κB signaling pathways in cigarette smoke–exposed cells and animal models [91].

LncRNAs provide an additional layer of regulation in COPD, modulating mRNA stability, transcription, protein scaffolding, and miRNA sponging [30]. In airway inflammation models, IL-6 Antisense RNA (1IL6-AS1) stabilizes IL-6 mRNA, thereby amplifying inflammatory signaling, while Long Antisense RNA of SP-A1 (LASI) increases mucin secretion and ICAM-1 expression in human epithelial cultures and patient-derived pulmonary organoids [91]. NQO1-AS1 reduces ROS production and maintains HMOX1 expression in bronchial epithelial cells exposed to cigarette smoke extract, protecting against oxidative stress [104]. LncRNAs also contribute to cellular remodeling processes: HOTAIR promotes endothelial apoptosis through DNMT1-mediated hypermethylation of the Bcl-2 promoter [97], whereas other lncRNAs, including CASC2, SNHG5, OIP5-AS1, and LUCAT1, act as competing endogenous RNAs, sequestering miRNAs to regulate apoptosis, inflammation, and airway remodeling in COPD [98, 99] (Table 2).

**Table 2:** Major epigenetic alterations involved in COPD pathogenesis and their clinical implications are reported

Epigenetic mechanism	Target/factor	Functional consequence in COPD	Refs
DNA methylation	AHRR locus	Modulation of smoking-related pathways	[69]
DNA methylation	ZDHHC14, NUB1, NLRP3, ZNF322	Dysregulated inflammatory and immune responses	[70]
DNA methylation	DNMT1	Gene silencing and chronic inflammation. Promotes promoter hypermethylation and transcriptional repression of key genes	[74]
DNA methylation	PTEN	Tumor suppressor activity	[75]
DNA methylation	PI3KCD	Modulates inflammatory signaling	[77]
DNA methylation	SERPINA1	Modulation of α1-antitrypsin expression and immune responses	[77]
Global DNA methylation	LINE-1 elements	Genomic instability	[77]
Environmental epigenetics	PRSS23	Altered airway gene regulation	[77]
Histone acetylation	HDACs	Increased inflammatory gene expression and corticosteroid resistance. Skeletal muscle dysfunction and cachexia	[81–83]
Histone methylation	PRMTs	Monocyte recruitment and cigarette smoke-induced lung injury	[87–89]
Histone phosphorylation	Histone H3	Chronic inflammation	[90]
miRNAs	Let-7 family	IL-6 production and myofibroblast differentiation	[92]
miRNAs	miR-181, miR-218, miR-155, miR-34a, miR-570	Increased NF-κB activation and cytokine release, enhanced inflammatory cytokine production. Influenced cellular senescence, impaired repair, remodeling	[94–100]
lncRNAs	IL6-AS1, LASI	Amplified inflammatory signaling	[91]
lncRNAs	HOTAIR, CASC2, SNHG5, OIP5-AS1, LUCAT1	Regulate apoptosis, inflammation and remodeling	[98, 99]
circRNAs	circRNA_0026344	Protection against epithelial apoptosis	[101, 102]
circRNAs	circBBS9, circ_0026466, circ_0006872	Increased inflammation and apoptosis	[91, 103]

In summary, ncRNAs, including miRNAs, lncRNAs, and circRNAs, integrate multiple regulatory signals in COPD, modulating inflammation, airway remodeling, senescence, and apoptosis, and represent potential targets for RNA-based diagnostics and therapeutics.

## Environmental Pollution in the Epigenetic Mechanisms of the Lung

Environmental contaminants exert profound effects on the epigenetic regulation of immune and inflammatory pathways, which play a central role in the pathophysiology of asthma and other chronic respiratory diseases. The lung, being continuously exposed to inhaled air, represents one of the primary physiological interfaces between the external environment and the human body. This unique anatomical and functional position makes the respiratory system particularly vulnerable to airborne pollutants. A wide range of contaminants, including fine particulate matter (PM2.5 and PM10), nitrogen oxides (NOx), ozone (O₃), volatile organic compounds (VOCs), polycyclic aromatic hydrocarbons (PAHs), heavy metals, and tobacco smoke, have been extensively documented as a major environmental risk factor for respiratory morbidity and mortality. Epidemiological evidence consistently link both acute and chronic exposure to these pollutants with the development and progression of asthma, and COPD [105, 106]. A growing body of epidemiological and experimental evidence demonstrates that exposure to ambient air pollution, particularly PM2.5, NOx, and O₃, is associated with alterations in DNA methylation patterns at both global and gene-specific levels. These epigenetic changes frequently affect genes involved in immune regulation, inflammation, xenobiotic metabolism, and cell cycle control, thereby contributing to disease susceptibility, progression, and the underlyining pathogenetic mechanism of respiratory disorders [107]. Notably, pollutant-induced epigenetic alterations have been detected in airway epithelial cells as well as in peripheral blood leukocytes, suggesting that air pollution exerts epigenetic effects at both the local and systemic levels in asthmatic subjects [37]. One of the most consistently reported epigenetic signatures of air pollution exposure is hypomethylation of repetitive DNA elements, such as long interspersed nuclear elements (LINE-1) and Alu sequences, particularly in children with airway diseases [108].

Global hypomethylation of repetitive DNA elements has important biological consequences. It can promote genomic instability, increased transposable element activity, and aberrant gene regulation, all of which are hallmarks of carcinogenesis. Indeed, reduced LINE-1 methylation has been associated with the progression of lung diseases and may represent an early molecular event induced by chronic exposure to air pollutants [109]. In contrast to global hypomethylation, environmental pollutants can also induce gene-specific hypermethylation, particularly in the promoter regions of genes involved in the regulation of cancerogenesis, affecting the progression of disease. Experimental studies using human bronchial epithelial cells have shown that exposure to diesel exhaust particles and cigarette smoke condensate leads to increased promoter methylation of genes such as CDKN2A (p16^INK4a^), MGMT, and RASSF1A. These epigenetic alterations have been observed in human bronchial epithelial cells cultured at the air–liquid interface, an experimental model that closely mimics the physiological conditions of airway exposure [110].

Promoter hypermethylation of these genes results in transcriptional silencing, thereby impairing critical cellular processes, including cell cycle regulation, DNA repair, and apoptosis. In the respiratory tract, this epigenetic repression contributes to the accumulation of genetic damage and facilitates serious cell transformation. Similar DNA methylation patterns have been observed in lung tissue and sputum samples from smokers and individuals exposed to high levels of ambient air pollutants, including PM2.5 and PM10, benzo[a]pyrene, NO₂/NOx, and black carbon, supporting the translational relevance of these in vitro findings [111]. Furthermore, epidemiological studies have reported altered DNA methylation of gene involved in cytokine signaling and innate immunity responses, including IFN-γ, IL6, and TLR4, in individuals exposed to traffic-related air pollution NO₂/NOx, PM2.5, PM10, ultrafine particles, black carbon, PAHs, carbon monoxide (CO), and VOCs [40, 112].

Specific components of air pollution, such as polycyclic aromatic hydrocarbons (PAHs) generated during combustion processes and constituents of tobacco smoke, have been shown to directly interfere with epigenetic programming through activation of the aryl hydrocarbon receptor (AHR) and modulation of DNA methyltransferase activity, thereby promoting inflammatory responses and carcinogenic pathways in lung tissue [113]. These data suggest that epigenetic dysregulation constitutes a critical mechanistic link between environmental exposures and the molecular alterations underlining inflammation, tissue remodeling, and disease progression.

Histone acetylation and methylation are dynamically regulated by histone-modifying enzymes and play a central role in controlling chromatin accessibility in response to environmental stressors. Environmental toxicants, such as phthalates and formaldehyde, exert profound effects on multiple epigenetic mechanisms, including global and gene-specific DNA methylation, chromatin remodeling, histone acetylation and methylation-dynamic RNA modifications, and the aberrant expression of miRNAs and lncRNAs. Through, these epigenetic, environmental toxicants modulate chromatin architecture and gene transcription, thereby driving the pathologenesis of a wide range of diseases, including airway disorders [114, 115].

Exposure to PM alters histone methylation marks, including H3K27me3 and H4K20me1, affecting the cellular stress responses, inflammatory signaling, and DNA damage response pathways. These epigenetic alterations contribute to epithelial dysfunction and airway remodeling in the lungs [112].

Many pollutants have been shown to modulate the expression or enzymatic activity of histone methyltransferases, including enhancer of zeste homolog 2 (EZH2) and euchromatic histone lysine methyltransferase 2 (EHMT2/G9a). These enzymes catalyze repressive histone marks such as H3K27me3 and H3K9me2, and play a key role in regulating cellular proliferation, senescence, and epithelial–mesenchymal transition (EMT). Dysregulated EZH2 and G9a activity has been observed following exposure to air pollution and has been associated with pathological airway remodeling, fibrotic responses, and malignant transformation in lung tissue. These findings suggest that pollutant-induced chromatin- alterations contribute to epithelial barrier dysfunction, immune imbalance, and progressive structural remodeling of the airways [112].

In addition to chromatin-level changes, ncRNAs represent a highly responsive regulatory layer linking environmental exposure to disease progression. Upregulation of pro-inflammatory and pro-oncogenic miRNAs, including miR-21 and miR-222, has been associated with urban air pollution exposure, whereas downregulation of miR-146a and miR-125b in smokers and individuals living in polluted environments amplifies Toll-like receptor and NF-κB signaling, thereby exacerbating airways inflammation and immune dysregulation[116].

Environmental epigenetics raises the possibility of transgenerational inheritance of pollutant-induced epigenetic changes. Preclinical animal studies shown that prenatal exposure to air pollution can cause persistent DNA methylation alterations in lung and immune cells, which may be transmitted to subsequent generations. These epigenetic changes have been associated with increased susceptibility to respiratory diseases, including asthma, through altered T-cell programming and dysregulated oxidative stress pathways. Although human evidence remains limited, emerging studies suggest that maternal exposure during pregnancy may impact offspring lung function and immune development, highlighting the need for further research on intergenerational effects [117].

Environmental factors, including race, ethnicity, and socioeconomic status, also influence DNA methylation patterns and asthma severity [118]. African American children with persistent asthma from low-income families exhibit higher global DNA methylation in blood cells, and in utero malnutrition increases asthma risk in offspring by altering the methylation patterns of Th2 cytokine genes [119]. These findings highlight how air pollution and other environmental agents can alter the epigenome, thereby increasing susceptibility to asthma.

Short- and long-term exposure to environmental pollutants, including traffic-related PAHs, PM2.5, vanadium, and gaseous pollutants as NO₂ and CO, can induce region-specific epigenetic modifications, including changes in DNA methylation of FOXP3 and IL-10 genes, thereby increasing susceptibility to asthma [120]. Transplacental exposure to pollutants can modify the methylation status of acyl-CoA synthetase long-chain family member 3 (ACSL3) in umbilical cord blood, increasing the risk of asthma in children under five years of age [121]. Furthermore, long-term exposure to pollutants such as NO₂ and PM2.5 has been associated with altered DNA methylation patterns in genes involved in asthma pathogenesis, including FOXP3, through effects on Treg cell function. Treg cells play a crucial role in asthma pathogenesis, by controlling immune responses and inflammation in the airways [120].

Other pollutants, such as black carbon and sulfates, also influence the methylation of genes involved in the immune regulation and asthma pathogenesis, including FCER1G (Fc fragment of IgE receptor Ig), IL4, and Ten-Eleven Translocation methylcytosine dioxygenase 1 (TET1). Exposure to bisphenol A (BPA) has been associated with specific DNA methylation patterns and correlated with asthma severity. Moreover, the toxicity of chemicals, such as BPA and phthalates is linked to epigenetic alteration affecting inflammation and Th2 immune responses, thereby increasing the asthma risk in children through changes in MAPK1 gene methylation [122]. Phthalate exposure has been shown to reduce TNF promoter methylation, increasing its expression and thereby elevating the risk of asthma [123]. Furthermore phthalates exposure may impair immune response, exacerbating allergies and asthma, and, in combination with indoor settled dust exposure, increase respiratory symptoms in children by reducing *TSLP* methylation [124]. Although the discussion so far has focused on the detrimental effects of environmental exposures, these influences should not be regarded as exclusively harmful. Environmental exposures may also induce protective epigenetic changes, with environmentally responsive epigenetic regulation promoting either pathogenic inflammation or adaptive, resilience-associated responses in chronic inflammatory disorders, depending on the nature, timing, and context of exposure [125]. In asthma, beneficial early-life exposure to microbially enriched environments has been associated with balanced immune maturation and reduced disease susceptibility, consistent with the hygiene hypothesis and its modern extensions [126]**.** Furthermore, accumulating evidence suggests that environmental biodiversity and host–microbe interactions promote immune tolerance and protect against allergic airway inflammation, supporting the concept that the pulmonary epigenome may mediate both beneficial environmental imprinting and disease-promoting epigenetic responses [127]. More strikingly, epigenetic changes are critical in regulating gene expression while leaving the DNA sequence unchanged. The interplay among genetic, environmental, and socioeconomic factors contributes to disease.

## Role of Epigenetic Modifications in the Senescence/Aging of the Lung

Aging is a major risk factor for chronic lung diseases, due to structural decline, weakened defence mechanisms, and epigenetic changes that affect gene expression. These processes contribute to cellular senescence, chronic inflammation, and progressive decline in lung function, thereby increasing susceptibility to disease. Aberrant DNA methylation patterns are associated with aging and allow chronological age to be estimated through epigenetic signatures. Accelerated epigenetic aging has been linked to increased disease risk and mortality, whereas slower epigenetic aging is associated with greater longevity [128]. Epigenetic clocks have shown that children with asthma aged 7 to 8 years, particularly those with allergic asthma, exhibit accelerated epigenetic aging, which is associated with higher IgE levels, FeNO, and increased risk of asthma and allergies [129].

One of the most compelling developments in aging research is the use of epigenetic clocks, which estimate biological age based on DNA methylation patterns at specific CpG sites. DNA methylation loci associated with changes in lung function have been shown to correlate with DNA methylation age and age acceleration difference (the difference between DNA methylation age and chronological age) [130]. Accelerated epigenetic aging has been observed in smokers and individuals with COPD, where it correlates with great disease severity and reduced lung function. In circulating immune cells, accelerated epigenetic aging reflects systemic immunosenescence and the chronic low-grade inflammation characteristic of aging [131]. These findings indicate that epigenetic age may serve both as a marker of lung health and as predictor of disease progression. By measuring biological age at the molecular level, epigenetic clocks provide a novel approach to assess how environmental and lifestyle factors influence lung aging and future disease risk [132].

Allergic asthma involves dysfunction of regulatory T cells (Tregs), which in patients exhibit a senescent and dysfunctional phenotype characterized by reduced suppressive capacity, increased inflammation, and decreased FOXP3 and IL-10 expression. Through a Dectin-1– and Raf-1/ROS–dependent signaling pathway, targeted epigenetic changes are induced, including active histone marks and DNA hypomethylation. These effects are lost in the absence of Dectin-1 [133].

Adolescent lung function can be predicted using epigenetic age acceleration (AA) combined with machine learning across multiple regression models. DNA methylation–based AAs were measured at ages 10 and 18 in 326 participants from the Isle of Wight Birth Cohort [134]. The results suggest that integrating AA tracking with machine learning may provide a useful approach for assessing lung cellular senescence, characterized by cell cycle arrest and a pro-inflammatory senescence-associated secretory phenotype (SASP). In the lung, stress-induced senescent cells accumulate over the time, leading to tissue damage, impaired repair, and chronic inflammation. Epigenetic alterations, particularly loss of DNA methylation at repetitive elements and global hypomethylation, further compromise genomic stability, promote inflammation, and contribute to disease progression in pulmonary disease [135].

Epigenetic modifications in senescent cells extend beyond global hypomethylation to include hypermethylation of specific gene promoters, particularly those regulating DNA repair and apoptosis regulator. This promoter hypermethylation silences protective genes, allowing damaged cells to persist and increasing the risk of malignant transformation, especially under chronic stress from factors like smoking and environmental pollution. These gene-specific methylation changes contribute to disease susceptibility by disrupting normal cell cycle regulation and apoptotic responses [135].

Histone modifications also play a key role in senescence, including increased levels of H3K9me3, a repressive mark, and decreased level of H3K27ac, an activating histone mark. These alterations promote chromatin compaction and transcriptional silencing of genes involved in cell proliferation and DNA repair. Consequently, senescent cells become less capable of responding to damage and inflammation, thereby further contributing to disease progression [136]. COPD models show a marked increase in histone lactylation, particularly at H4 lysine 12 (H4K12la). Cleavage Under Targets and Tagmentation (CUT&Tag) sequencing has revealed that H4K12la regulates the CD38–NAD⁺ pathway, promoting senescence of type II alveolar epithelial cells (AEC2) and driving COPD progression [137]. The Polycomb group factor EZH2, an H3K27 methyltransferase, is downregulated in senescent cells, promoting senescence through two mechanisms: (a) rapid activation of the DNA damage response in proliferating cells, and (b) gradual loss of H3K27me3, which induces CDKN2A (p16) expression and activates SASP genes. Depletion of H3K27me3 may acts as a molecular “timer” that coordinates the transition from DNA repair to stable senescence. EZH2 activity is regulated by WNT and MYC signaling, as well as by DNA damage–induced protein turnover, providing further insight into the molecular mechanisms of senescence during aging [138].

A “redox clock” was developed and validated in healthy individuals, including both males and females. Age-related changes in NRF2 promoter methylation were observed, with stronger effects in males. The model demonstrated the ability to detect accelerated biological aging through changes in NRF2 promoter methylation patterns. [139]. H3K9me2, mediated by histone methytransferase G9a, plays a key role in regulating the dynamics of lung epithelial progenitor cells, although this control declines with age. In the aged lungs, reduced H3K9me2 leads to a decrease in AT2 progenitors and impaired alveolar regeneration, while enhancing the activity of bronchiolar progenitor cells and promoting repair. Overall, aging disrupts the epigenetic coordination of lung repair, thereby increasing susceptibility to chronic lung disease [140]. Finally, aging affects lung structure and physiology, as well as the biology of immune system cells, thereby contributing to the development of respiratory diseases including asthma and COPD through multiple cellular and molecular mechanisms. These findings underscore the need for personalized interventions and therapeutic strategies. Future research should focus on identifying and validating reliable aging biomarkers and age-related disease phenotypes to improve risk stratification and guide precision medicine approaches [141].

## Conclusions and Perspectives

Recent advances have clarified the role of epigenetic mechanisms in inflammatory airway diseases, showing that they mediate gene–environment interactions and influence inflammation, tissue remodeling, and cellular transformation. This conceptual framework describes how epigenetic mechanisms, including DNA methylation, histone modifications, and ncRNAs regulate key molecular and cellular processes involved in chronic respiratory diseases such as asthma and COPD. Dysregulation of these processes contributes to chronic inflammation, immune imbalance, epithelial dysfunction, and disease progression. The dynamic and reversible nature of epigenetic mechanisms also provides opportunities for clinical applications, particularly through the development of non-invasive biomarkers detectable in airway samples, blood, or exhaled breath, useful for diagnosis, prognosis, and treatment monitoring. Today epigenetic therapies are increasingly being explored for lung diseases. Additionally, the use of miRNA mimics and antagomirs, which modulate post-transcriptional gene expression, holds potential for targeted treatments in conditions such as inflammation and fibrosis. More recently, have emerged as promising approaches to precisely reprogram disease-associated epigenetic marks, potentially offering improved specificity and reduced off-target effects compared with earlier techniques [142].

Looking ahead, key research directions aim to translate epigenetic knowledge into clinical practice. A major focus is the integration of multi-omics approaches combining epigenomics, transcriptomics, proteomics, and metabolomics to better characterize gene–environment interaction networks driving disease, enabling the identification of novel biomarkers and therapeutic targets [143]. Longitudinal cohort studies are also essential to clarify causal links between epigenetic changes and disease onset or progression, supporting the development of early diagnostic markers and effective interventions. Overall, personalized epigenetic medicine may improve the management of chronic respiratory diseases by tailoring therapies to each patient’s genetic and environmental profile.

## Data Availability

No datasets were generated or analysed during the current study.
